# The Bio-Tribological Effect of Poly-Gamma-Glutamic Acid in the Lysozyme-Ionic Contact Lens System

**DOI:** 10.3390/polym12010156

**Published:** 2020-01-07

**Authors:** Chen-Ying Su, Lung-Kun Yeh, Chi-Chun Lai, Mihaela Dubuisson, Yi-Fei Tsao, Ching-Li Tseng, Hsu-Wei Fang

**Affiliations:** 1 Department of Chemical Engineering and Biotechnology, National Taipei University of Technology, 1, Sec. 3, Zhongxiao E. Rd., Taipei 10608, Taiwan; chenying.su@gmail.com (C.-Y.S.); susan84103086@gmail.com (Y.-F.T.); 2 Department of Ophthalmology, Chang Gung Memorial Hospital, Linkou. No. 5, Fuxing St., Taoyuan 333, Taiwan; yehlungkun@gmail.com (L.-K.Y.); chichun.lai@gmail.com (C.-C.L.); 3 College of Medicine, Chang Gung University, No.259, Wenhua 1st Rd., Taoyuan 333, Taiwan; 4 Anton Paar TriTec SA, Les Vernets 6, 2035 Corcelles-Cormondrèche, Switzerland; mihaela.dubuisson@anton-paar.com; 5 Graduate Institute of Biomedical Materials and Tissue Engineering, College of Biomedical Engineering, Taipei Medical University, No. 250, Wu-Hsing St., Taipei 110, Taiwan; chingli@tmu.edu.tw; 6 Institute of Biomedical Engineering and Nanomedicine, National Health Research Institutes, No. 35, Keyan Road, Zhunan Town, Miaoli County 35053, Taiwan

**Keywords:** bio-tribology, poly-gamma-glutamic acid, lysozyme, ionic contact lens

## Abstract

Feeling comfortable is an important issue for contact lens wearers as contact lenses are worn for an extensive period of time. It has been shown that the in vitro friction coefficient of contact lenses is correlated to the degree of in vivo comfort, thus many studies focus on establishing friction testing methods for investigating the friction coefficient of contact lenses or contact lens care solution. We have previously demonstrated the lubricating property of poly-gamma-glutamic acid (γ-PGA)-containing care solution, and it could reduce the high friction coefficient caused by lysozyme. However, the mechanism of how γ-PGA-containing care solution reduces the lysozyme-induced friction coefficient of contact lenses is unclear. We investigated the bio-tribological effect of γ-PGA on ionic contact lenses in the presence of lysozyme by testing load and velocity variations. The ability to remove lysozyme deposition by γ-PGA and viscosity analysis of γ-PGA-containing care solutions were also investigated to understand the potential mechanism. Our results showed that the friction coefficient of γ-PGA-containing care solution with lysozyme was the lowest in both load and velocity variations, and γ-PGA functions distinctly in the lysozyme-ionic contact lens system. We proposed a model of how γ-PGA could reduce the friction coefficient in these two conditions.

## 1. Introduction

Many myopia patients choose to wear contact lenses for convenience and appearance in addition to correct vision. Feeling comfortable becomes a critical factor for contact lens wearers due to an extensive wearing period. Once contact lenses are worn, some tear film components such as proteins are immediately deposited on the surface of the lens. When tear proteins accumulate on the contact lens, immune reactions can be triggered resulting in discomfort, red eyes, or even eye conditions such as conjunctivitis [1,2]. Therefore, removing deposited proteins from the lens or preventing proteins from being adsorbed becomes a critical function for contact lens care solutions along with other functions such as rinsing, inhibiting microbial growth, and storing contact lenses. 

It has been shown that the in vitro friction coefficient of contact lenses is corresponded to an in vivo comfort degree [3,4], thus modifying the materials or the surface of contact lenses has been investigated for reducing friction coefficient of contact lenses [5]. Non-functionalized polyvinyl alcohol (PVA) is added to Nelfilcon-A lenses during the manufacturing process to provide comfort. Non-functionalized PVA from Nelfilcon-A lenses has been proved to enhance comfort feeling, and even help to reduce dryness for some contact lens wearers [6,7]. Another good way for providing lubrication is by adding lubricants to contact lens care solutions. Lubricants in the care solution might be kept on the surface of the lens during the storage time, thus wearers may feel comfortable when wearing contact lenses the next day. Hyaluronan (HA) is a commonly used lubricant in contact lens solutions because of its high hydrophilic properties and viscoelastic nature [8].

Poly-gamma-glutamic acid (γ-PGA) is a naturally occurring polymer and it has been applied in the medicine, cosmetics, and sanitary industry [9,10]. Our previous study has shown that γ-PGA can be a lubricant in the contact lens care solution, especially it could reduce the high friction coefficient caused by lysozymes [11,12]. However, the potential mechanism is still unclear. Therefore, we investigated the bio-tribological effects of γ-PGA on lysozyme-ionic contact lens in this study. One of the ionic contact lenses, Etafilcon-A, was used. Different normal loads and velocities were tested to investigate the effects of γ-PGA in the lysozyme-ionic contact lens system. In addition, the viscosity property and the ability to remove adsorbed lysozyme of γ-PGA were investigated to understand the potential mechanism of γ-PGA as a lubricant. Our results demonstrated that the interactions between γ-PGA and lysozyme were distinct under the conditions of different normal loads and velocities, and provided a bio-tribological prove of γ-PGA with functions of removing adsorbed lysozyme and providing lubrication.

## 2. Materials and Methods

### 2.1. Chemicals, Reagents, and Contact Lens

To prepare 100 mL of γ-PGA-containing contact lens care solution, 0.015 g CaCl_2_ (Sigma, St. Louis, MO, USA), 0.15 g KCl (Sigma, St. Louis, MI, USA), 0.45 g NaCl (Sigma, St. Louis, MI, USA), 1.8 g Na_2_HPO_4_ (Sigma, St. Louis, MI, USA), and 0.5 g ethylenediaminetetraacetic acid (EDTA, Sigma) were added into 100 mL of distilled water. 1.5 g of γ-PGA (Vedan Enterprise Corporation, Taichung City, Taiwan) and 0.05 g poloxamer-407 (Wei Ming Pharmaceutical Mfg. Co., Ltd., Taipei City, Taiwan) were then added into the solution, and the solution was passed through a 0.22 μm filter (EMD Millipore Corporation, Billerica, MA, USA). 40 μL of 25 mg/mL epigallocatechin gallate (EGCG, Sigma), 0.1 mL of hyaluronic acid (HA, Maxigen Biotech Inc., Taoyuan City, Taiwan), and 2.5 mL of 5% chlorine dioxide (ClO_2_, Sigma) were added into the filtered solution. For comparing the friction coefficient of different solutions, 1 DAY ACUVUE MOIST contact lens (Etafilcon-A, Johnson & Johnson, New Brunswick, NJ, USA) was used. Lysozyme powder (Sigma, St. Louis, MI, USA) was dissolved in either phosphate-buffered saline (PBS) or γ-PGA-containing contact lens care solution for the final concentration as 1.9 mg/mL.

### 2.2. In Vitro Contact Lens Friction Testing System

A Nano Tribometer (NTR^3^, Anton Paar, Graz, Austria) was used for testing the friction coefficient of different solutions. The testing was conducted under a linear reciprocating movement with an amplitude of 2 mm and the contact lens was sliding against a ruby ball with a radius of 2 mm. Contact lenses were removed from the package, and the excess liquid from the lens was removed by lens tissue. The contact lens was mounted onto a semi-spherical sample holder with a plastic base that matches the internal curvature of the lens. The lens was clamped with the upper part of the holder. Three magnetic pegs are embedded in the clamping upper part and in the support lower part of the contact lens holder. Two types of tests were performed in this study. For the load variations, the speed was set to 0.2 cm/s while the load varied between 0.4 and 5 miniNewton (mN). For the speed variations, the load was set to 1 mN while the speed varied between 0.03 and 1 cm/s. There were 50 sequences for each test and 30 cycles for each sequence. For example, 1 sequence represented when the lens was sliding at 0.2 cm/s under 0.4 mN for 30 cycles. The next sequence would be the lens was sliding at 0.2 cm/s for 30 cycles under 0.5 mN if it was for the load variations. All tests were performed at room temperature and the contact lenses were completely immersed in the tested solutions during the entire time.

### 2.3. Lysozyme Concentration Measurement

The Bio-Rad DC protein assay (Bio-Rad, Hercules, CA, USA) was used and the lowest limit of this assay was 5 μg/mL. The preparation was done according to the manufacturer’s instructions. The standard curve was established by preparing 0, 0.05, 0.1, 0.2, 0.4, 0.8, 1.6, and 3.2 mg/mL of lysosomal solutions. After the reaction was completed, each sample was read by an Enzyme-Linked ImmunoSorbent Assay reader with a wavelength of 750 nm, and the optical density value was obtained. The lysozyme concentration in the sample could be obtained by using the standard curve.

### 2.4. Lysozyme Deposition Analysis

Etafilcon-A contact lenses were placed in 1.9 mg/mL lysozyme solution (original solution) at 37 °C for 8, 16, or 24 h. The lens was then transferred to γ-PGA-containing contact lens care solution, and was on the shaker for 30 min and then removed. Each condition was repeated four times. Lysozyme concentration in the original solution and in the care solution was measured after the lens was removed, thus lysozyme deposition concentration on each lens was calculated as below:

[1.9 − (lysozyme concentration in the original solution) − (lysozyme concentration in the care solution)]

### 2.5. Viscosity Analysis

The viscosity of solutions was measured by DV-III Ultra programmable rheometer (Brookfield, Middleboro, MA, USA). The speed was 6 rpm (revolution per minute), and the temperature was at 25 °C. The viscosity of γ-PGA-containing care solution with and without lysozyme was measured, and each sample was repeated 3 times.

### 2.6. Statistical Analysis

Differences in lysozyme deposition analysis and viscosity analysis between different conditions were assessed by the student’s *t*-test to make an allowance of comparisons. A value of *p* < 0.05 was considered significant.

## 3. Results

### 3.1. The Effect of Normal Loads on Bio-Tribological Characteristics of γ-PGA

**Figure 1:**
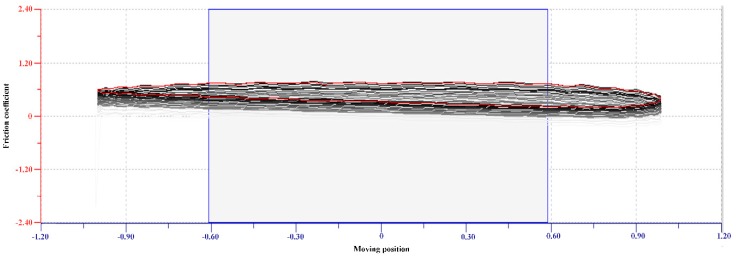
The raw data of the friction coefficient of one condition. The raw data is taken from 1 sequence which is under 0.4 mN load and 0.2 cm/s when the Etafilcon-A lens is sliding in phosphate buffer solution (PBS) for 30 cycles. The *x*-axis shows Etafilcon-A is moving between the position of −1.0 mm and 1.0 mm (amplitude of 2 mm), and the *y*-axis shows the friction coefficient. The rectangle box represents the middle of the cycles, and values of friction coefficient in the rectangle box are averaged as the friction coefficient for this sequence.

**Figure 2:**
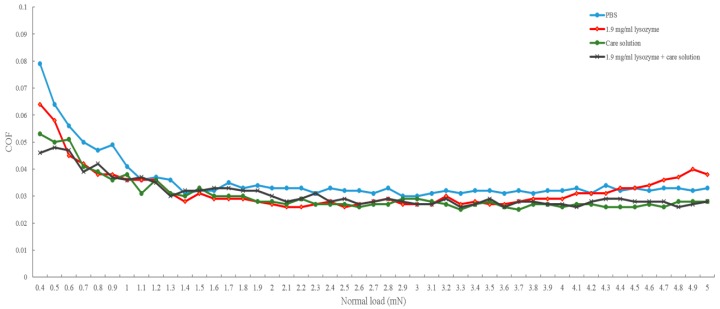
The comparison of the friction coefficient of different solutions for load variations. The friction coefficient of PBS (blue line), 1.9 mg/mL lysozyme solution (red line), γ-PGA-containing care solution (green line), and 1.9 mg/mL lysozyme and γ-PGA-containing care solution (black line) when normal load varies from 0.4 mN to 5 mN at velocity of 0.2 cm/s.

The raw data of the friction coefficient curve was shown in Figure 1 as an example, the lens was sliding in PBS at 0.2 cm/s when the normal load was 0.4 mN. Figure 1 represented the friction coefficient values of 30 cycles in one sequence. The values acquired around the middle of each cycle were considered for the cycle-averaged value for 1 sequence. Figure 2 demonstrated the friction coefficient of the lens in different solutions. The friction coefficient of PBS was the highest until the normal load was larger than 4.4 mN and the friction coefficient of the lysozyme solution was then the highest until the normal load reached 5 mN. The friction coefficient of lysozyme, the γ-PGA-containing care solution, and the combination of the lysozyme and care solution were very close when the normal load was smaller than 4 mN. When the normal load was larger than 4 mN, the friction coefficient was lower if the solution contained γ-PGA (Figure 2). Overall, the friction coefficient of each solution was decreased when the normal load was increased.

### 3.2. The Effect of Velocities on Bio-Tribological Characteristics of γ-PGA

**Figure 3:**
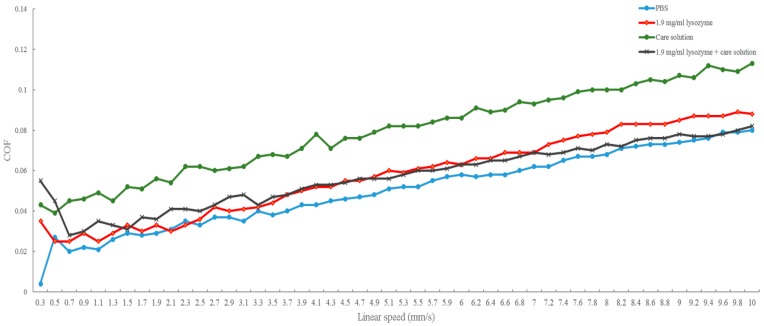
The comparison of the friction coefficient of different solutions for velocity variations. The friction coefficient of PBS (blue line), 1.9 mg/mL lysozyme solution (red line), γ-PGA-containing care solution (green line), and 1.9 mg/mL lysozyme and γ-PGA-containing care solution (black line) when velocity varies from 0.3 mm/s to 10 mm/s at normal load of 1 mN.

In contrast, the friction coefficient of each solution increased when the velocity increased (Figure 3). Unlike the result of load variations, the friction coefficient of γ-PGA-containing care solution was the highest while PBS represented the lowest. The friction coefficient of the lysozyme solution and the combination of lysozyme and care solution was similar when the velocity was slower than 7 mm/s. When the velocity was faster than 7 mm/s, the friction coefficient of lysozyme was higher than that of the lysozyme solution and γ-PGA-containing care solution (Figure 3).

### 3.3. The Physical Properties of γ-PGA-Containing Care Solution

**Figure 4:**
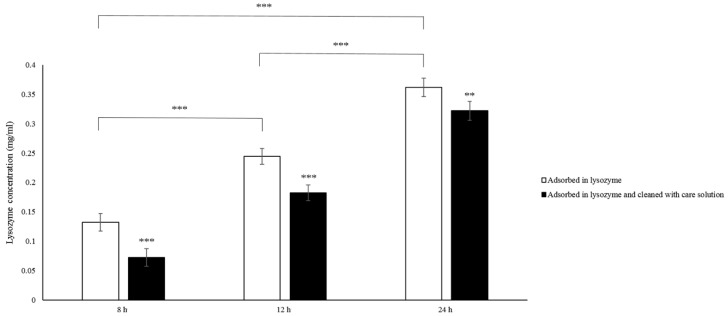
Lysozyme deposition with and without being cleaned by the γ-PGA-containing care solution. The deposited lysozyme concentrations are measured directly after the Etafilcon-A lenses are placed in 1.9 mg/mL lysozyme solution for 8, 16, or 24 h (white bars), or after being cleaned by γ-PGA-containing care solution (black bars). ** *p* < 0.01 and *** *p* < 0.001.

To understand the potential bio-tribological mechanism of γ-PGA in the lysozyme-ionic contact lens system, lysozyme removal ability and viscosity of γ-PGA-containing care solution were investigated. After the lens was incubated in lysozyme, the amount of lysozyme deposition increased significantly when the incubation time was longer (Figure 4). After washing the lens with the γ-PGA-containing care solution, the amount of lysozyme deposition was significantly reduced. The concentration of lysozyme deposition was from 0.13 to 0.07, from 0.24 to 0.18, and from 0.36 to 0.32 mg/mL for 8, 16, and 24-h incubation time, respectively. When the incubation time was shorter, the lysozyme was easier to be removed by the γ-PGA-containing care solution (Figure 4).

The averaged viscosity of the γ-PGA-containing care solution was 11.94 cP (centipoise), while the average viscosity of the combination of γ-PGA-containing care solution and lysozyme was 12.62 cP. The viscosity of the combination increased significantly (*p* < 0.01) compared with the γ-PGA-containing care solution only.

## 4. Discussion

In this study, we investigated the bio-tribological effect of γ-PGA-containing care solution in the lysozyme-ionic contact lens system. When the normal load varied from 0.4 to 5 mN, the friction coefficient of PBS was the highest until the normal load was larger than 4.4 mN and then the friction coefficient of lysozyme was the highest. When the solution contained γ-PGA, the friction coefficient was the lowest overall. When the velocities varied from 0.03 and 1 cm/s, the friction coefficient of γ-PGA-containing care solution was the highest. The friction coefficient of lysozyme and the combination of lysozyme and γ-PGA-containing care solution was similar, but the friction coefficient of lysozyme became higher when the velocity was faster than 0.7 cm/s. The lysozyme deposition result demonstrated that the γ-PGA-containing care solution could effectively remove the adsorbed lysozyme from Etafilcon-A contact lenses. In addition, the viscosity analysis showed that adding lysozyme would increase the viscosity of the γ-PGA-containing care solution.

**Figure 5:**
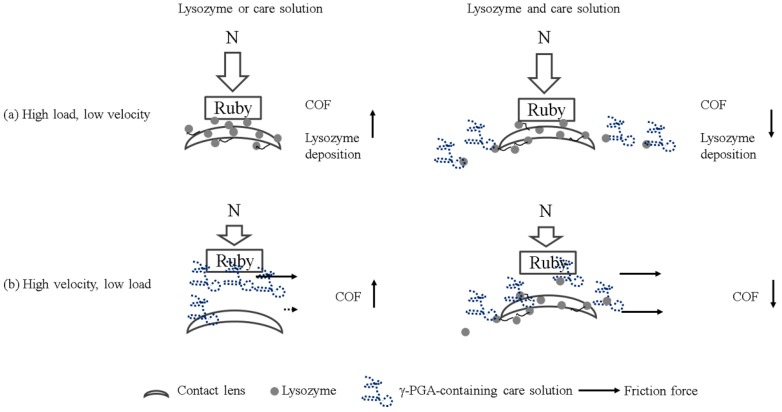
The potential mechanism of the bio-tribological effect of γ-PGA in the lysozyme-ionic contact lens system. (**a**) When Etafilcon-A is sliding against a ruby ball under a high normal load, lysozyme deposition is increased resulting in an increase of friction coefficient (**left**). When γ-PGA is present, γ-PGA can bring lysozyme away from the surface resulting in a reduction of both lysozyme deposition and friction coefficient (**right**). (**b**) When Etafilcon-A is sliding under fast velocity, γ-PGA interacts differently with Etafilcon-A and with ruby resulting in an uneven friction force on two surfaces. The differential friction forces result in an increase in friction coefficient (**left**). In contrast, the interaction between γ-PGA and lysozyme may be stronger resulting in an even friction force on two surfaces. Therefore, the friction coefficient of γ-PGA-containing care solution and lysozyme is reduced in this condition (**right**).

Multiple lubrication regimes have been shown to exist during the blinking cycle when contact lenses are in the eye [13]. When the load is high and the blinking speed is slow, boundary lubrication occurs between the contact lens and the eyelid. Under boundary lubrication, the lubricant between two sliding surfaces forms a thin layer. Whether the lubricant can be adsorbed on the surface determines the friction coefficient, rather than the viscosity of the lubricant [14]. Therefore, the result of load variations could be explained by boundary lubrication (Figure 5a). The friction coefficient of the lysozyme was higher than the friction coefficient of γ-PGA-containing care solution and of the combination of lysozyme and γ-PGA-containing care solution (Figure 2). The lysozyme deposition result demonstrated that the amount of lysozyme deposition was increasing when the incubation time was longer (Figure 4), thus the amount of lysozyme on the surface of Etafilcon-A might be increased during frictional movement. In addition, the water content of lens material also affects protein deposition [15,16]. Etafilcon-A is a high water content material resulting in larger pore sizes, thus the lysozyme would penetrate into the matrix of Etafilcon-A. Therefore, the friction coefficient of lysozyme was higher on the end of load variation testing due to the high amount of adsorbed lysozyme. When γ-PGA was present with lysozyme, γ-PGA might take away lysozyme from the lens surface resulting in a reduction of friction coefficient (Figure 5a). Lysozyme was positively charged while both γ-PGA and Etafilcon-A were negatively charged [17,18], thus γ-PGA could take away lysozyme by forming an ionic bond. However, why lysozyme would bind to γ-PGA instead of Etafilcon-A requires further investigation.

When the load is low and the blinking speed is fast, elastohydrodynamic or hydrodynamic lubrication occurs between the contact lens and the eyelid during blinking [13]. At high speed, the lubricant with high viscosity would separate two surfaces and shear stress increases resulting in a high friction coefficient. The result of velocity variations might be explained by elastohydrodynamic or hydrodynamic lubrication. The viscosity of 1.9 mg/mL lysozyme was around 0.8 cP [19], and it was lower than the γ-PGA-containing care solution resulting in a lower friction coefficient (Figure 3). Interestingly, the viscosity of lysozyme and γ-PGA-containing care solution was higher than the viscosity of the γ-PGA-containing care solution but the friction coefficient was smaller (Figure 3). This result suggested that the materials of surfaces and the interaction between γ-PGA and contact surfaces should be taken into account. When ruby is in acid solution, hydrogen bonds can be adsorbed on the surface resulting in a positively charged surface [20]. We then hypothesized that negatively charged γ-PGA would bind to the ruby surface but would be away from negatively charged pHEMA surface. The adhesion affinity of γ-PGA to two surfaces might result in different friction forces, resulting in an increase of friction coefficient (Figure 5b). In contrast, the presence of lysozyme may interact with γ-PGA resulting in similar friction force between the γ-PGA/ruby surface and γ-PGA/pHEMA surface. Therefore, the friction coefficient of γ-PGA-containing care solution and lysozyme was smaller than the γ-PGA-containing care solution (Figure 3).

Although we proposed a potential model for the effect of γ-PGA in the lysozyme-ionic contact lens system under load and velocity variations, there are some limitations. We have not yet verified whether the amount of lysozyme deposition was indeed increased on the end of frictional cycles, or whether γ-PGA and lysozyme could form a stronger ionic bond than the bond between lysozyme and Etafilcon-A. In addition, we need to investigate whether γ-PGA would interact differently with ruby and with the Etafilcon-A lens to better understand the bio-tribological mechanism for velocity variations. 

The current study demonstrated that γ-PGA can reduce the high friction coefficient caused by lysozyme in load variations. In velocity variations, the combination of γ-PGA and lysozyme showed a lower friction coefficient although the friction coefficient of γ-PGA itself was high. However, whether the in vitro results can be applied for increasing comfort degree in vivo requires further investigation.

## 5. Conclusions

We investigated the bio-tribological effect of γ-PGA in the lysozyme-ionic contact lens system. The friction testing of load and velocity variations demonstrated distinct results. In load variations, the friction coefficient of lysozyme was the highest while the friction coefficient of γ-PGA-containing care solution and the combination of lysozyme and γ-PGA-containing care solution were the lowest at larger normal loads. In velocity variations, the friction coefficient of γ-PGA-containing care solution was the highest while lysozyme and the combination of lysozyme and γ-PGA-containing care solution showed a lower friction coefficient at higher velocities. The lysozyme deposition analysis demonstrated γ-PGA could effectively remove adsorbed lysozyme from the lens, suggesting that the interaction between γ-PGA and lysozyme might be strong thus the friction coefficient of γ-PGA-containing care solution and lysozyme was the lowest in friction testing. Therefore, our data provided a potential contact lens care solution that can both clean ionic contact lenses effectively and provide lubrication in the presence of lysozyme.
